# Battlefield Acupuncture for the Treatment of Chronic Migraines

**DOI:** 10.7759/cureus.60369

**Published:** 2024-05-15

**Authors:** Niyaz Uddin, Diane L Levine

**Affiliations:** 1 Internal Medicine, Wayne State University School of Medicine, Detroit, USA

**Keywords:** complementary and alternative medicine (cam), acupuncture, neurology, general internal medicine, battlefield acupuncture, migraine treatment, migraine disorder, primary headache disorder, chronic migraine (cm)

## Abstract

A 70-year-old man presented with worsening migraines and was referred to a neurologist by their primary care doctor for further workup. Imaging and lab work were benign. The patient then underwent several trials of various first and second-line medications and anti-migraine devices to no avail. It was not until one session of battlefield acupuncture, where five needles were placed in the patient’s ear for a few days, that the patient had a resolution of his symptoms.

## Introduction

Battlefield acupuncture (BFA) is a form of auricular acupuncture originally developed by Dr. Richard Niemtzow in 2001 for military members to reduce acute and chronic pain symptoms [1]. It involves the placement of five semipermanent needles in the ears at specific locations according to the somatotopic organization of the body represented in the auricle that was standardized in 1957 by French neurologist Dr. Paul Nogier [2]. The needles then remain in the patient’s ear for several days until falling out on their own. This non-pharmacologic intervention can be learned by non-acupuncturist healthcare providers, can be quickly and easily administered, can have immediate effects [3,4], and can be effective with repeated use [4]. While its mechanism remains to be fully understood, it has been shown to be an effective treatment for chronic pain and, as presented in this report, for chronic migraines as well.

## Case presentation

**Figure 1:**
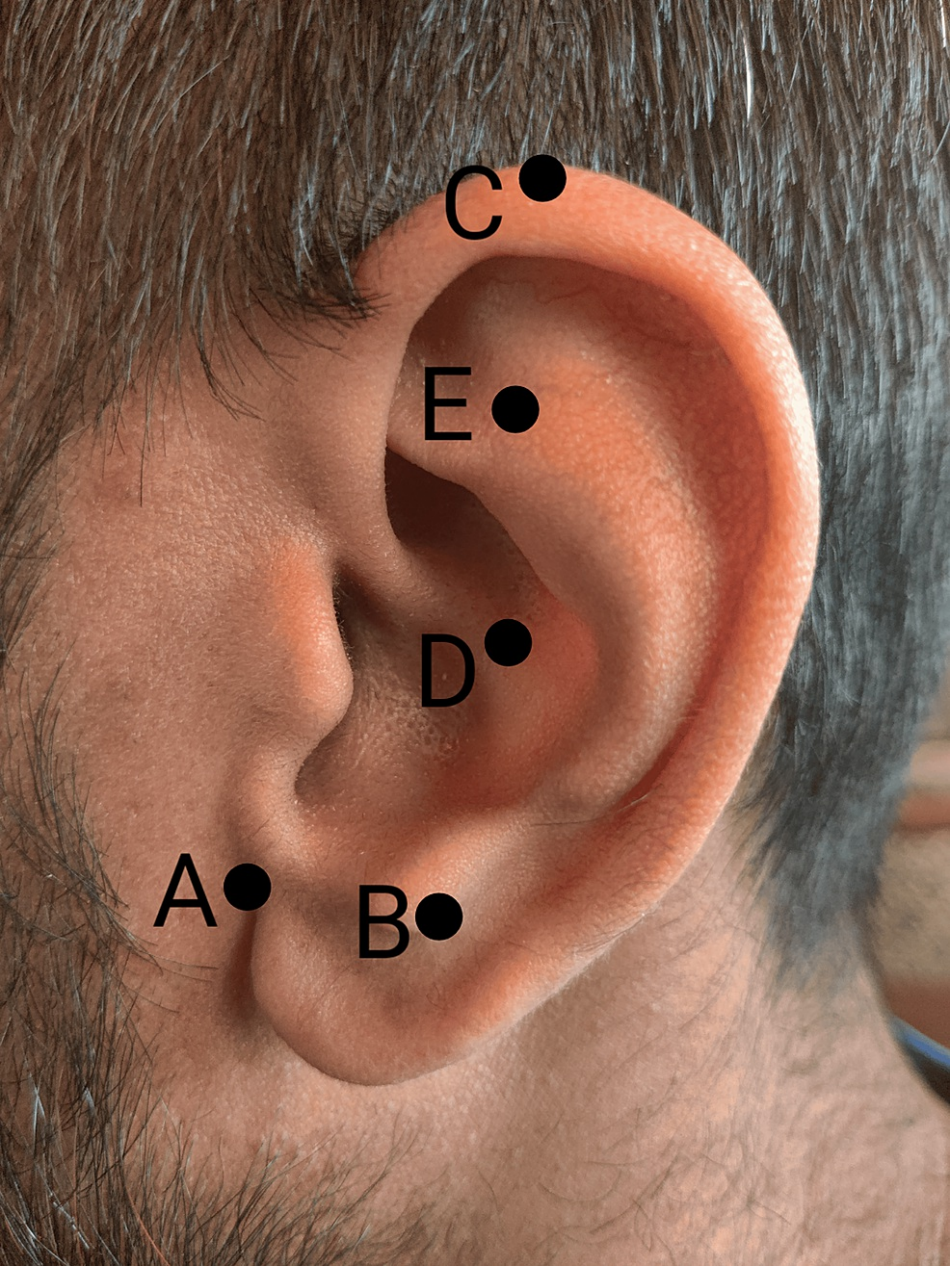
The left ear with the prescribed acupuncture points for battlefield acupuncture labeled in the order in which they are placed: (A) cingulate gyrus, (B) thalamus, (C) omega 2, (D) point zero, and (E) Shen Men. [5].

A male in his early 70s presented to his primary clinic for worsening migraines. He did not have any other significant medical history that was found to be contributory to his current presentation. The patient had a history of episodic migraines since 1970 after sustaining a traumatic brain injury during military service. Within the last few weeks, however, his migraines had progressed to chronic migraines as they increased in frequency and severity to the point that they occurred anywhere from three days a week to daily, and at times were unbearably severe. He had to leave his work due to the severity. His migraines were not associated with any auras, sensory changes, visual issues, or balance problems. He did endorse feeling irritable, having to lie down in a dark room, photophobia, and phonophobia. He revealed his symptoms had been refractory to treatments in the past, including topiramate and sumatriptan. The patient used to manage his episodic migraines with aspirin and caffeine, but these were no longer working for him. The patient was referred to neurology for further workup. Neurology ordered an MRI which showed no abnormalities that could explain his migraines. The patient was also given a trial of a Cefaly device, Nerivio, rizatriptan, and propranolol over the course of a few follow-ups with neurology. None of these measures led to any significant relief. The patient was also suggested to try Botox treatment, but he refused. However, he was amenable to a trial of acupuncture as this facility contained a suite dedicated to holistic treatment modalities. The locations where the five needles were placed can be seen in Figure 1. The entire procedure took less than five minutes. The needles were placed in the patient’s left ear, per practitioner and patient preference. The patient reported feeling slight discomfort upon insertion of the needles. He was then sent home and instructed to allow the needles to fall out on their own, which for this patient took almost a week. An interview with the patient regarding his experience with BFA is included in the Appendix. The patient presented to the clinic three weeks after his first trial of BFA and reported a resolution of his symptoms. The patient had not had a full-blown migraine since treatment. He did endorse occasionally feeling a migraine come on but said this was rare and would quickly subside. The patient elected to continue receiving BFA in lieu of other pharmacological therapies.

Following up with the patient seven months later, he had not done BFA for the last three months as the severity and frequency of his migraines had significantly decreased after the first treatment to a manageable level allowing him to return to his regular life. For maintenance of his now episodic migraines, he was using aspirin and caffeine which had been effective in the past when his symptoms were milder. According to the patient, the other treatments he was given for his migraines offered little, if any, benefit to him. BFA, in his words, offered immediate relief, and after the first session, the benefits lasted approximately three weeks. The patient underwent BFA three more times afterward but stated it was not as effective but still helpful. The patient was referred to try a full-body acupuncture course once a week for eight weeks but did not find it beneficial.

## Discussion

Migraines affect over one billion people per year worldwide and can have debilitating effects on people’s careers, parenting, relationships, and the economy [6]. Chronic migraines (more than 15 migraines per month), specifically, affect about 2% of the world’s population [7]. The most recent results of the Global Burden of Disease study in 2019 continue to list headache disorders as one of the leading causes of disability [8]. This illustrates the widespread burden of migraines and the necessity to consider all possible treatment modalities, especially ones such as BFA, which are affordable, low risk, easy to train and administer, and can avoid rebound headaches from medication overuse. Additionally, the side effects are minimal. The most commonly reported adverse effects are pain at the needle insertion site and lightheadedness [9].

Relatively recent systematic reviews and meta-analyses [9-11] showcase how sparse studies on BFA are and highlight that many of them are not conclusive due to factors such as small sample size, study design, and potential biases. However, this did not stop the United States Department of Defense from investing millions into teaching BFA to healthcare providers in the military and at Veterans Affairs hospitals throughout the country [1].

While BFA is generally described as a potential treatment for acute and chronic pain and has mostly been studied in that capacity [9-11], this case illustrates the utility of BFA as a treatment modality for chronic migraines as well, especially when it is refractory to other treatments. There are a handful of studies that look at auricular acupuncture specifically in treating migraine-type symptoms [12] with conflicting results. BFA, as of now, is still poorly studied and requires much more research to further elucidate its mechanism, indications, dosage, and frequency, as well as its use in acute and chronic pain, musculoskeletal and neuropathic pain, and other types of headaches [13]. Furthermore, thus far, it is generally only accessible to the veteran population through veterans’ health centers and most studies have relied on data extrapolated from this group. With further investigation and more robust data, BFA should be offered as an adjunct or alternative treatment for many conditions such as migraines to the general public; particularly, in the context of more patients leaning toward integrative medicine [14].

## Conclusions

BFA is a form of auricular acupuncture where five semi-permanent needles are placed in the ear and has historically been used by the military for the treatment of chronic pain; however, its uses may extend beyond that. Chronic migraines are a debilitating illness and can cause a great burden on a patient’s day-to-day life. Although BFA requires more research, it is a promising alternative treatment for migraines that is non-pharmacological, easy to learn and administer, and carries few risks.

## Appendices

The patient had the following to say regarding their treatment with BFA:

I had heard of acupuncture and that’s what I assumed I was getting. Lay down and they stick some needles in your back or whatever. I was really surprised when he told me what it was. Putting them in your ear and leaving them in there. That was a total surprise. I was not a believer, but I was willing to try. I felt great. I didn’t have a headache. Didn’t have one for about three or four weeks. Living a normal life. I was a much nicer person to be around. I’ve been telling everybody about it. There are quite a few people I know that have them, not in the military. I told a friend of mine’s wife, about the same age as me, who’s getting it done and it’s been very effective for her. I would highly suggest it. I’m not sure it works for everybody, but it definitely worked for me. It wasn’t painful. It was just a little sharp prick when they stick it in the earlobe or whatever. The most painful was when you would take a shower or something and try to dry your ears and it would hang up on the little needles. That was kind of uncomfortable. Sleeping on them was a little uncomfortable. But nothing that you couldn’t handle. It was about a week for the needles to fall out. And you don’t even see them or feel them. Just a little tiny gold dot on the end of the needle. They just stay in there. If you look in the mirror you might be able to see they’re not there anymore.
